# Morphological and molecular characterization of *Karyolysus* – a neglected but common parasite infecting some European lizards

**DOI:** 10.1186/s13071-014-0555-x

**Published:** 2014-12-10

**Authors:** Božena Haklová-Kočíková, Adriana Hižňanová, Igor Majláth, Karol Račka, David James Harris, Gábor Földvári, Piotr Tryjanowski, Natália Kokošová, Beáta Malčeková, Viktória Majláthová

**Affiliations:** Institute of Parasitology, Slovak Academy of Sciences, Hlinkova 3, 040 01 Košice, Slovak Republic; Institute of Biology and Ecology, University of P. J. Šafárik in Košice, Moyzesova 11, 040 01 Košice, Slovak Republic; Department of Parasitology, State Veterinary Institute Jihlava, Rantířovská 93, 586 05 Jihlava, Czech Republic; CI-BIO-UP, Centro de Investigação em Biodiversidade e Recursors Genéticos da Universidade do Porto, Campus Agrário de Vairão, 4485-661 Vairão, Portugal; Department of Parasitology and Zoology, Faculty of Veterinary Science, Szent István University, 2 István Street, Budapest, H-1078 Hungary; Institute of Zoology, Poznań University of Life Sciences, Wojska Polskiego 71 C, 60-625 Poznań, Poland; Department of Biochemical Sciences, Charles University in Prague, Faculty of Pharmacy in Hradec Kralove, Hradec Kralove, Czech Republic

**Keywords:** *Karyolysus*, *Ophionyssus*, Lacertidae, lizards, Europe

## Abstract

**Background:**

Blood parasites of the genus *Karyolysus* Labbé, 1894 (Apicomplexa: Adeleida: Karyolysidae) represent the protozoan haemogregarines found in various genera of lizards, including *Lacerta, Podarcis*, *Darevskia* (Lacertidae) and *Mabouia* (Scincidae). The vectors of parasites are gamasid mites from the genus *Ophionyssus*.

**Methods:**

A total of 557 individuals of lacertid lizards were captured in four different localities in Europe (Hungary, Poland, Romania and Slovakia) and blood was collected. Samples were examined using both microscopic and molecular methods, and phylogenetic relationships of all isolates of *Karyolysus* sp. were assessed for the first time. *Karyolysus* sp. 18S rRNA isolates were evaluated using Bayesian and Maximum Likelihood analyses.

**Results:**

A total of 520 blood smears were examined microscopically and unicellular protozoan parasites were found in 116 samples (22.3% prevalence). The presence of two *Karyolysus* species, *K. latus* and *K. lacazei* was identified. In total, of 210 samples tested by polymerase chain reaction (PCR), the presence of parasites was observed in 64 individuals (prevalence 30.5%). Results of phylogenetic analyses revealed the existence of four haplotypes, all part of the same lineage, with other parasites identified as belonging to the genus *Hepatozoon*.

**Conclusions:**

Classification of these parasites using current taxonomy is complex - they were identified in both mites and ticks that typically are considered to host *Karyolysus* and *Hepatozoon* respectively. Furthermore although distortions to the intermediate host erythrocyte nuclei were observed, the defining characteristic of *Karyolysus*, the haplotypes were nearly identical to those reported from lizards in the Iberian Peninsula, where such distortions were not reported and which were thus identified as *Hepatozoon*. Based on the phylogenetic analyses, neither vertebrate host, nor geographical patterns of the studied blood parasites could be established.

**Electronic supplementary material:**

The online version of this article (doi:10.1186/s13071-014-0555-x) contains supplementary material, which is available to authorized users.

## Background

Reptiles often serve as hosts for unicellular blood parasites belonging to the suborder Adeleorina, mainly from the genera *Haemogregarina*, *Hepatozoon* and *Karyolysus. Haemogregarina* was found in various species of terrapins, while *Hepatozoon* is the typical apicomplexan parasites found in different species of snakes and lizards [1], each distinguished by very different developmental patterns in their invertebrate hosts in which sporogony occurs. To date, *Karyolysus* has been reported mainly in European lizards [2-5], as well as in Asia [6]. The genus *Karyolysus* Labbé 1894 includes ten currently recognized species: *K. lacertae* Danilewsky, 1886, *K. lacazei* Labbé, 1894, *K. biretortus* Nicolle, 1904, *K. berestnewi* Finkelstein, 1907, *K. bicapsulatus* Franca, 1910, *K. zuluetai* Reichenow, 1920, *K. subtilis* Ricci, 1954, *K. octocromosomi* Alvarez-Calvo, 1975, *K. latus* Svahn, 1975 and *K. minor* Svahn, 1975.

The life cycle of *Karyolysus* sp. is indirect; merogony occurs in an intermediate vertebrate host, while gamogony and sporogony takes place in the gut of an invertebrate final host [1]. Gamasid mites *Ophionyssus* sp. Oudemans, 1901, belonging to the order Mesostigmata, act as the main vectors. These are strictly obligate parasites and can utilize hosts that are taxonomically related (lizards, snakes) [7,8]. *Karyolysus* transmission to the lizard is thought to involve swallowing mites containing infectious sporozoites, without typical sporocystic arrangements. Meronts can be observed in capillary endothelium of the liver, lungs, heart, and spleen, while gamonts parasitize erythrocytes in peripheral blood of lizards [1,4]. While *Hepatozoon* is transmitted by a wide spectrum of invertebrates, including hard ticks, transmission of *Karyolysus* sp. from infected sand lizard (*Lacerta agilis)* to larvae and nymphs of *Ixodes ricinus* ticks was not experimentally demonstrated [4], although ticks on these lizards are more abundant than mites [9]. *Karyolysus* represent well defined group different in morphology and in life cycle, it differs from the closely related genera *Hepatozoon* and *Hemolivia* in several characteristics of its biology. In the life cycle, motile sporokinetes are formed in oocyst by a single germinal center and are released in host organism and encyst as sporocyst in *Karyolysus*. The genus *Hepatozoon* is characterized by a large polysporocystic oocyst. Intraerytrocytic merogony occurs in *Hemolivia*, moreover gamonts in the peripheral blood have typical morphology with the presence of a stain-resistant vacuole.

Although *Karyolysus* includes ten known species, only few authors classified parasites found in lizards to the species level [2-5]. Information about morphometry and morphology of few *Karyolysus* species are available [10], and species determination is very difficult and ambiguous: moreover measurements of cell size and area ratios may be modified due to alternative preparations of blood smears [3]. Therefore several authors classified detected parasites only as “haemogregarines” or “blood parasites” [9,11-14]. The presence of blood parasites was detected in various European lizards including *Algyroides nigropunctatus*, *Iberolacerta horvathi*, *Podarcis muralis* and *P. melisellensis* from Austria and Croatia [3], *Podarcis lilfordi* from Balearic Islands [11], *L. agilis* and *Zootoca vivipara* from Poland, Denmark and Sweden [4,5,9], *L. viridis* from Hungary [15], *Podarcis bocagei* and *Podarcis carbonelli* from Portugal [16], *L. agilis chersonensis* from Romania [2] and *Iberolacerta monticola*, *P. muralis* and *Timon lepidus* from Spain [12-14].

Molecular data of parasites found in erythrocytes of European lizards are scarce. Only a few publications exist where molecular method were used to detect blood parasites in European lizards (*Algyroides marchi, P. bocagei*, *P. hispanica* and *P. lilfordi*); using phylogenetic analysis parasites from these hosts were identified as *Hepatozoon* sp., since they were nested within this group and since little or no distortion of the vertebrate host erythrocyte nucleus was observed, the defining characteristic of *Karyolysus* [17,18]. Molecular data of *Karyolysus* are not available in GenBank and the phylogenetic position of this genus in comparison to other reptile parasites is not known yet. Therefore it is necessary to obtain molecular data of *Karyolysus*, not only from lizards but also from *Ophionyssus* sp. mites, which are the only known vector of this parasite. *Karyolysus* parasites are neglected in molecular and phylogenetic analyses. More molecular data are available for *Hemolivia*, however the phylogenetic position of the genus remains unclear [19-21].

To summarize, the aim of the present study was to detect the presence of *Karyolysus* sp. in lizards and their ectoparasites, and to determine the taxonomy and phylogenetic relationship of parasites in various species of lizards from several regions of Europe (Hungary, Poland, Romania and Slovakia) using both molecular and microscopic examination methods. In this way we aim to test if different genetic lineages of parasites occur in various regions in Europe, since considerable diversity has been identified in blood parasites in other reptiles from this region [22].

## Methods

### Study areas

**Figure 1:**
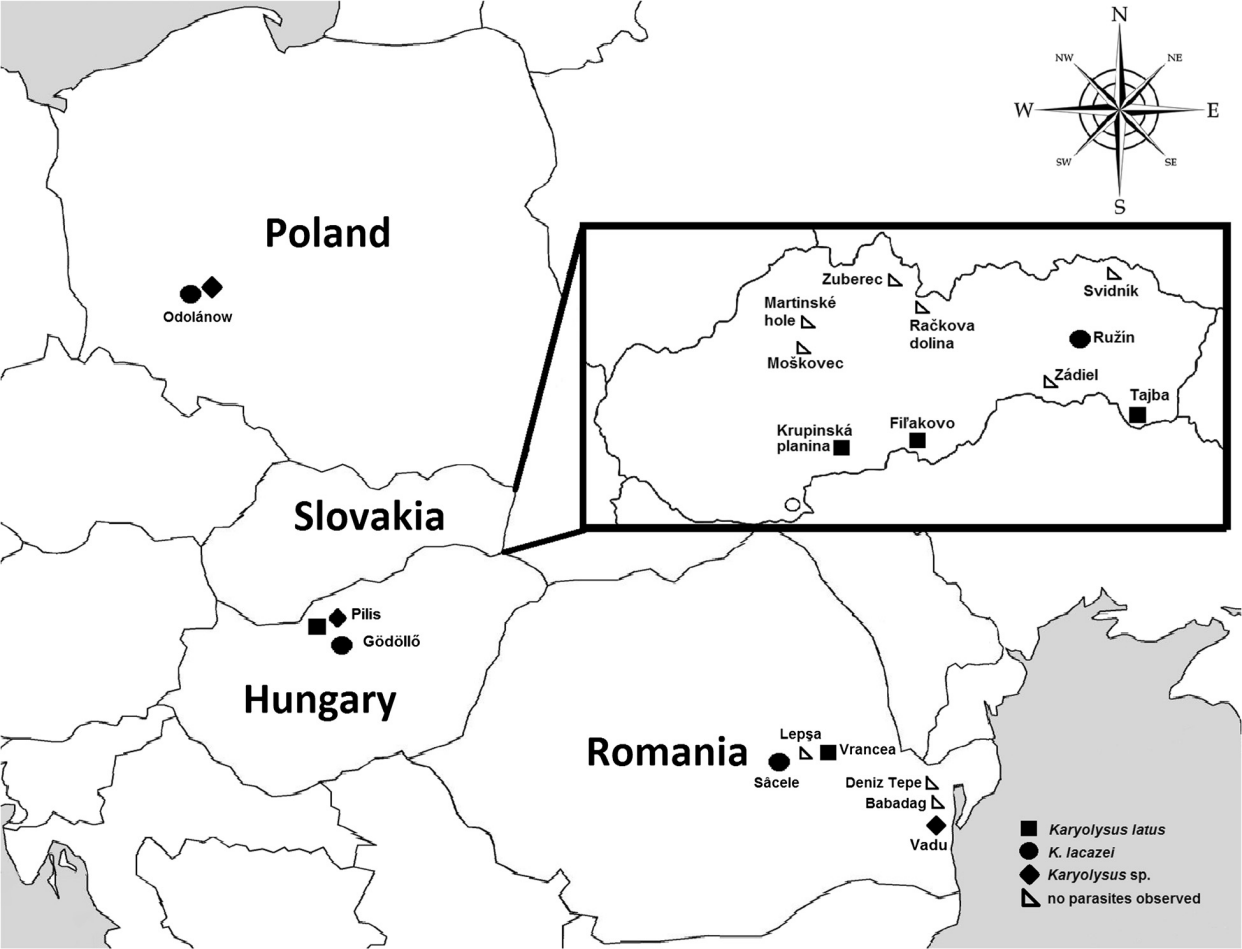
**Localities in Europe of capturing of lizards; filled dots indicate localities with incidence of blood parasites, empty dots localities where blood parasites were not detected.**

Biological samples from lizards were collected during field expeditions, which were undertaken in four countries in Europe (Poland, Hungary, Romania and Slovakia) (Figure 1, Table 1). Field trips were carried out from 2004 to 2013.

**Table 1:** **Lizard species examined in this study**

**Species**	**Country**	**Study area**	**Captured individuals**	**Microscopy examined/infected**	**PCR examined/infected**	**Species of** ***Karyolysus***
*Lacerta agilis*	Poland	Odolanów	33	8/3	32/10	*K. lacazei*
	Slovakia	Tajba	1	1/0	1/0	*-*
		Svidník	16	16/0	16/0	*-*
		Martinské hole	11	11/0	-	*-*
		Račkova dolina	4	4/0	-	*-*
		Moškovec	4	4/0	-	*-*
	Romania	Lepşa	5	5/0	5/1	*-*
*L. agilis* ssp. *exigua*	Romania	Vadu	20	20/2	20/11	*Karyolysus* sp.*
*L. agilis* ssp. *erythronota*	Romania	Vadu	1	1/0	1/1	*-*
*L.viridis*	Slovakia	Burda	91	91/0	24/0	*-*
		Zádiel	136	136/0	14/0	-
		Tajba	36	36/12	9/1	*K. latus*
		Krupinská planina	2	2/0	2/1	*-*
	Hungary	Gödöllő	69	69/52	36/17	*K. lacazei*
		Pilis	7	7/1	7/3	*Karyolysus* sp.*
	Romania	Babadag	3	3/0	3/1	*-*
*L. viridis* ssp. *meridionalis*	Romania	Deniz Tepe	1	1/0	1/0	*-*
*L. trilineata dobrogica*	Romania	Sâcele	10	10/6	10/5	*K. lacazei*
*Zootoca vivipara*	Poland	Odolanów	16	5/0	11/3	*Karyolysus* sp.
	Slovakia	Ružín	1	1/1	-	*K. lacazei*
		Zuberec	17	17/0	-	*-*
		Račkova dolina	11	11/0	-	*-*
		Martinské hole	2	2/0	-	*-*
	Romania	Vrancea	2	2/2	1/0	*K. latus*
*Podarcis muralis*	Slovakia	Krupinská planina	39	39/26	10/10	*K. latus*
		Fiľakovo	10	10/10	-	*K. latus*
	Hungary	Pilis	9	8/1	7/0	*K. latus*
**TOTAL**		**20**	**557**	**520/116**	**210/64**	**-**
**PREVALENCE**				**22.3%**	**30.5%**	**-**

For each species, the total number of individuals tested, infected and species of parasites found are given, using microscopy or through PCR amplification. Asterisks indicate smears, where identification of parasite species was unsuccessful because of low parasitemia observed.

The first locality was near the town of Odolanów, Poland (51° 34’N, 17° 40’E). This area is characterized by intensively farmed land with a mosaic of arable fields, meadows, and small woodlots and scattered trees and shrubs of different age.

The second locality is in Hungary near the town of Gödöllő (47° 36’N, 19° 22’E) and the mountain Pilis (47° 41’N, 18° 52’E). These localities are characteristic by maple oak and lime oak callow forests with bushes separated by less-covered moorlands; stone-pit, shady groves, protected natural values as well as in the south and in the east part of area vineyards are very common.

Reptiles in Romania were captured in various areas including Sâcele (45° 37’N, 25° 42’E) typically by abandoned irrigation canals as a part of the steppe biogeographical region; Vadu (44° 26’N, 28° 44’E) with vegetation represented by perennial shrubs, tall grass and species of rush (*Juncus* sp.), reed (*Phragmites* sp.) and bulrush (*Typha* sp.); Lepşa (45° 56’N, 26° 34’E) situated in the valley of Lepșa river, where vegetation is represented by hydrophilous tall herb fringe communities, alluvial groves with *Alnus* sp. and patches of grass; Babadag (44° 53’N, 28° 20’E) located on a small lake formed by the Taiţa river, in the densely wooded highlands of northern Dobruja; Deniz Tepe (44° 59’N, 28° 41’E), locality which is represent by hill at an elevation of 163 meters above sea level and Vrancea (45° 48’N, 27° 04’E), seismically active area, over 11% of the country surface covered with vine and located in elevation of 170 meters above sea level.

Finally, in Slovakia lizards were captured in various habitats including bog communities (Tajba, 48° 26’N, 21° 46’E), mountain areas (Zuberec 49° 18’N, 19° 36’E; Martinské hole 49° 08’N, 18° 49’E; Račkova dolina 49° 05’N, 19° 47’E; Moškovec 48° 59’N, 18° 49’E), castle ruins (Fiľakovo 48° 15’N, 19° 49’E), areas near the water basin (Ružín 48° 55’N, 21° 02’E), xerothermous karst areas (Zádiel 48° 39’N, 20° 56’E), xerothermous areas of volcanic origin (Burda 47° 52’N, 18° 54’E and Krupinská planina 48° 13’N, 19° 05’E) as well as peripheral areas of city agglomeration (Svidník 49° 20’N, 21° 33’E).

### Sample collection

Lizards were captured by noosing or by hand. Blood from lizards was taken via a ventral puncture of the *vena coccygea*. Blood for molecular analysis was stored in tubes with sodium citrate. In case of tail loss, tail tips were stored in 70% ethanol.

Ticks and mites were collected from lizards immediately in the field and stored in 70% ethanol. Some individuals were kept in white linen bags during the night. In the morning, engorged mites had left the lizards and were collected from the bags. Animals captured in the field were released after sampling at the capture place. The mites were kept in test-tubes in the laboratory (23°C, 80% air humidity).

### Slide examination

Smears from mites and their eggs were performed in the laboratory, air dried, fixed with methanol and stained with a Giemsa solution (30 minutes) and evaluated under a light microscope.

Blood smears from lizards were made and air-dried immediately in the field. In the laboratory staining was performed using May-Grünwald (10 minutes) and Giemsa solution (30 minutes) and examined with a light microscope at × 400 magnifications. Approximately 50 microscopic fields on each smear were examined for the presence of blood parasites. When no parasites were detected by this method, the smear was considered negative. Mean length (Ml) and mean width (Mw) of various parasite stages and their nuclei found in positive smears were measured at × 1000 magnifications.

A total of 520 individuals comprising of 8 species of lizards were examined for blood parasites. Details are given in Table 1.

### DNA extraction, amplification and sequencing

DNA isolation (blood or tissue) was carried out using a commercial kit (NucleoSpin Blood and Tissue, Macherey-Nagel, Düren, Germany) according to the manufacturer’s protocol. Isolated DNA was stored at - 20°C.

PCR reactions were run in a 25 μl reaction mixture from the Taq DNA Polymerase kit (Qiagen, Hilden, Germany) containing 2.5 μl 10xPCR Coral Load PCR Buffer (15 pmol/μl MgCl_2_); 1 μl MgCl_2_ (25 pmol/μl); 0.5 μl dNTPs (10 pmol/μl); 0.5 μl of each primer (10 pmol/μl) (Integrated DNA Technologies, Leuven, Belgium); 0.125 μl *Taq* DNA Polymerase (5 U/μl); 14.875 μl water for molecular biology (Water, Mol Bio grade DN-ase, RN-ase, and Protease-free; 5Prime, Hamburg, Deutschland) and 5 μl of DNA. Verification that the isolated DNA was appropriate for PCR amplification was assessed using primers, which amplify the 12S rRNA [23]. Molecular detection of blood parasites was made by PCR reactions with HEPF300 (5’ GTT TCT GAC CTA TCA GCT TTC GAC G 3’)/HEP900 (5’ CAA ATC TAA GAA TTT CAC CTC TGA C 3’) [24] and HEMO1 (5’ TAT TGG TTT TAA GAA CTA ATT TTA TGA TTG 3’)/HEMO2 (5’ CTT CTC CTT CCT TTA AGT GAT AAG GTT CAC 3’) [25] primers targeting part of the 18S rRNA gene. The prepared mix was preheated to 95°C for 5 min. Amplification with HEP300/HEP900 primers was performed as described by Ujvari et al. [26], but with an annealing temperature of 51°C, while annealing temperature with HEMO1/HEMO2 primers was set to 48.8°C.

In each PCR reaction negative (Water, Mol Bio grade DN-ase, RN-ase, and Protease-free; 5Prime, Hamburg, Deutschland) and positive (already sequenced sample) controls were included. Amplicons were separated on a 1.5% agarose gel (Sigma-Aldrich, Buchs, Switzerland) in 1 × TAE Buffer (40 mM Tris, pH 7.8, 20 mM acetic acid, 2 mM EDTA). The gel was stained by Good View nucleic acid stain (Ecoli, Bratislava, Slovak republic) and afterwards was visualized using a UV transilluminator. Obtained positive PCR products (approximately 600 bp) were purified by GenElute™ PCR Clean-Up Kit (Sigma-Aldrich, Buchs, Switzerland) and sequenced by a commercial sequencing facility (University of Veterinary Medicine, Košice, Slovak republic), with all fragments sequenced in both directions.

### Phylogenetic analyses

Sequences were visualized, edited using MEGA 4 and checked by eye. Checked sequences were compared to the sequences available in GenBank by using the basic local alignment search tool (BLASTn 2.2.26) and all of them matched with sequences of *Hepatozoon* sp. from various hosts. Based on Tomé *et al.* [22], related *Hepatozoon* sequences were downloaded and aligned using Clustal W. The final alignment consisted of 93 individuals, with 584 bps.

Maximum Likelihood (ML) analysis with random sequence addition was used to estimate evolutionary relationships using PhyML [26]. Support for nodes was estimated using the bootstrap technique [27] with 100 replicates. The model of evolution employed was chosen using the AIC criteria carried out in Modeltest 3.06 [28]. Bayesian analysis was implemented using Mr. Bayes v.3.2 [29] with parameters estimated as part of the analysis. The analysis was run for 10,000,000 generations, saving one tree every 1,000 generations. The log-likelihood values of the sample point were plotted against the generation time and all the trees prior to reaching stationary were discarded as burn-in samples. Remaining trees were combined in a 50% majority consensus tree. *Haemogregarina balli* Paterson and Desser, 1976 and *Dactylosoma ranarum* Lankester, 1882 were used as outgroups following Barta *et al.* [19].

To facilitate visualization of the phylogenetic relationships within the lineage including our isolates, a network was made using a region of 455bp of isolates from this lineage. The network was produced using a Median-Joining analysis with default parameters in software Network 4.6.1.0 [30].

## Results

### Microscopic examination

A total of 520 blood smears representing 4 species and 4 subspecies of lizards from different localities, were examined: 381 samples from Slovakia (36 *Lacerta agilis*, 265 *L. viridis*, 31 *Zootoca vivipara* and 49 *Podarcis muralis*), 84 from Hungary (76 *L. viridis* and 8 *P. muralis*), 42 from Romania (5 *L. agilis*, 20 *L. agilis* ssp. *exigua*, 1 *L. agilis* ssp. *erythronota*, 3 *L. viridis*, 1 *L. viridis* ssp. *meridionalis*, 10 *L. trilineata* ssp. *dobrogica* and 2 *Z. vivipara*) and 13 from Poland (8 *L. agilis* and 5 *Z. vivipara*).

The presence of protozoan parasites localized in red blood cells was observed in 116 samples, including 3 *L. agilis* from Poland, 2 *L. agilis* ssp. *exigua* from Romania, 12 *L. viridis* from Slovakia, 53 *L. viridis* from Hungary, 6 *L. trilineata* ssp. *dobrogica* from Romania, one *Z. vivipara* from Slovakia, 2 *Z. vivipara* from Romania, 36 *P. muralis* from Slovakia and one *P. muralis* from Hungary (22.3% prevalence) (Table 1).

Two species of *Karyolysus*, *K. latus* and *K. lacazei*, were identified based on morphology, measurements of the of parasite, as well as measurements of the parasite’s nuclei as described by Svahn [4].

**Figure 2:**
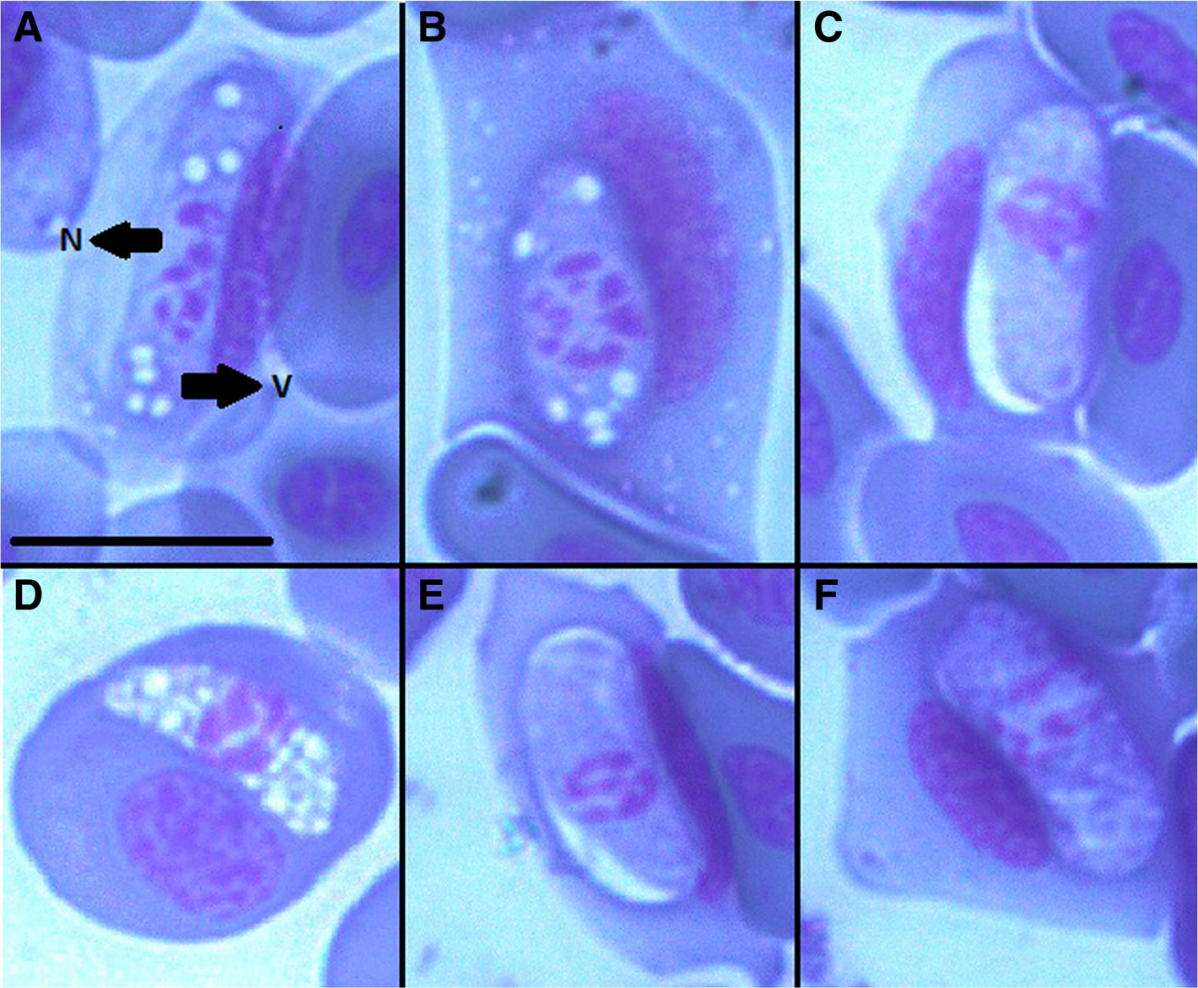
***Karyolysus latus*** **A-B and D trophozoites in blood smear of** ***Podarcis muralis*** **, C and E-F gamonts, C and E microgamonts, F macrogamonts.** Arrows indicates organelles: V = vacuoles, N = nucleus, Ma = macrogamonts, Mi = microgamonts; scalebar = 10 μm.

*K. latus* was found to infect *P. muralis* in Krupinská planina and *L. viridis* from Tajba. Trophozoites (Ml 11.40μm, Mw 5.10μm) found in blood smears were oval shaped, lentiform or beanshaped with pale vacuolated cytoplasm and a large diffuse reticulated centrally placed nucleus (Ml 3.80 μm, Mw 4.20 μm) (Figure 2, A-B and D). Gamonts were also oval with rounded ends with non-vacuolated cytoplasm. A distinct space was observed surrounding the parasite within the red blood cell (Figure 2, C and E). Cytoplasm of macrogamonts (Ml 11.53 μm, Mw 4.83 μm) stained dark blue, with a diffuse nucleus (Ml 3.63 μm, Mw 4.10 μm), located centrally (Figure 2, F). Microgamonts (Ml 11.93 μm, Mw 4.97) stained light blue and the nuclei (Ml 3.63 μm, Mw 4.07 μm) were more compact (Figure 2, C and E). There is no capsule surrounding the gamonts.

Host cells were hypertrophied and their nuclei were displaced by the parasite. In most cases the nuclei of the host cells were elongated and compressed, pushed to one of the long sides of the parasite, sometimes displaced to one of the ends of parasite (Figure 2).

**Figure 3:**
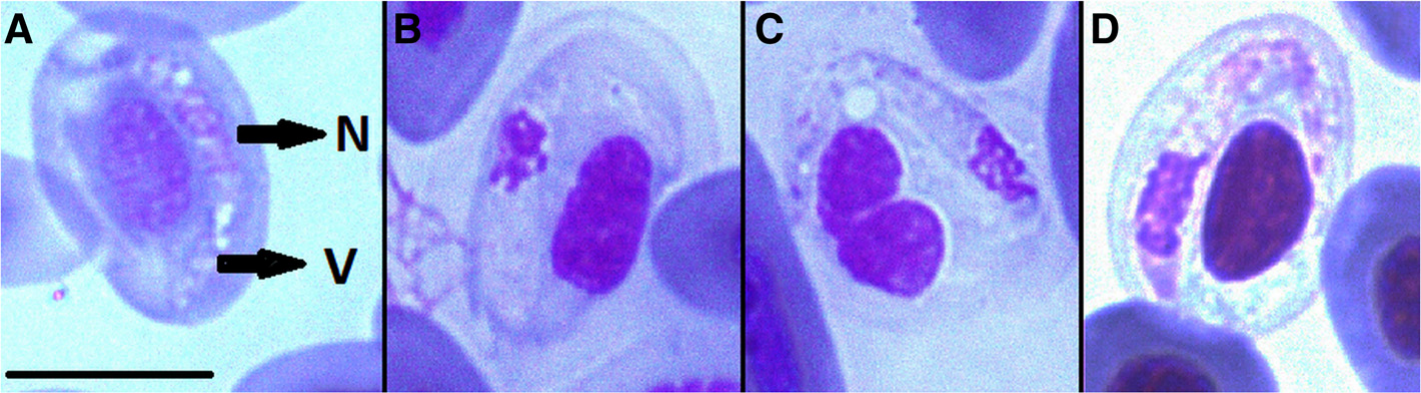
***Karyolysus lacazei*** **A trophozoite found in blood smear of** ***Lacerta trilineata*** **, Romania, B-D gamonts found in** ***Lacerta viridis*** **from Hungary.** Arrows indicates organelles: V = vacuoles, N = nucleus; scalebar = 10μm.

*K. lacazei* was identified in *L. agilis* in Poland, *L. viridis* in Hungary and *L. trilineata* ssp. *dobrogica* in Romania. Trophozoites are thin and elongated, and the cytoplasm is vacuolated (Figure 3, A). It was not possible to distinguish micro and macrogamonts. The shape of the cells is slender and thin with one end bent. The cytoplasm of gamonts (Ml 20.69 μm, Mw 2.8 μm) stained pale blue, and vacuoles were not present. The position of the nucleus (Ml 4.68 μm, Mw 2.54 μm) is shifted laterally, or placed at the distal end of the parasite (Figure 3, B - D). The presence of the parasite caused great changes in the appearance of the host cells, which was hypertrophied with cytoplasm observed with difficulty (loss of staining properties). The nuclei of infected hosts were swollen, sometimes compressed and darkly stained (Figure 3).

### Ectoparasites

**Figure 4:**
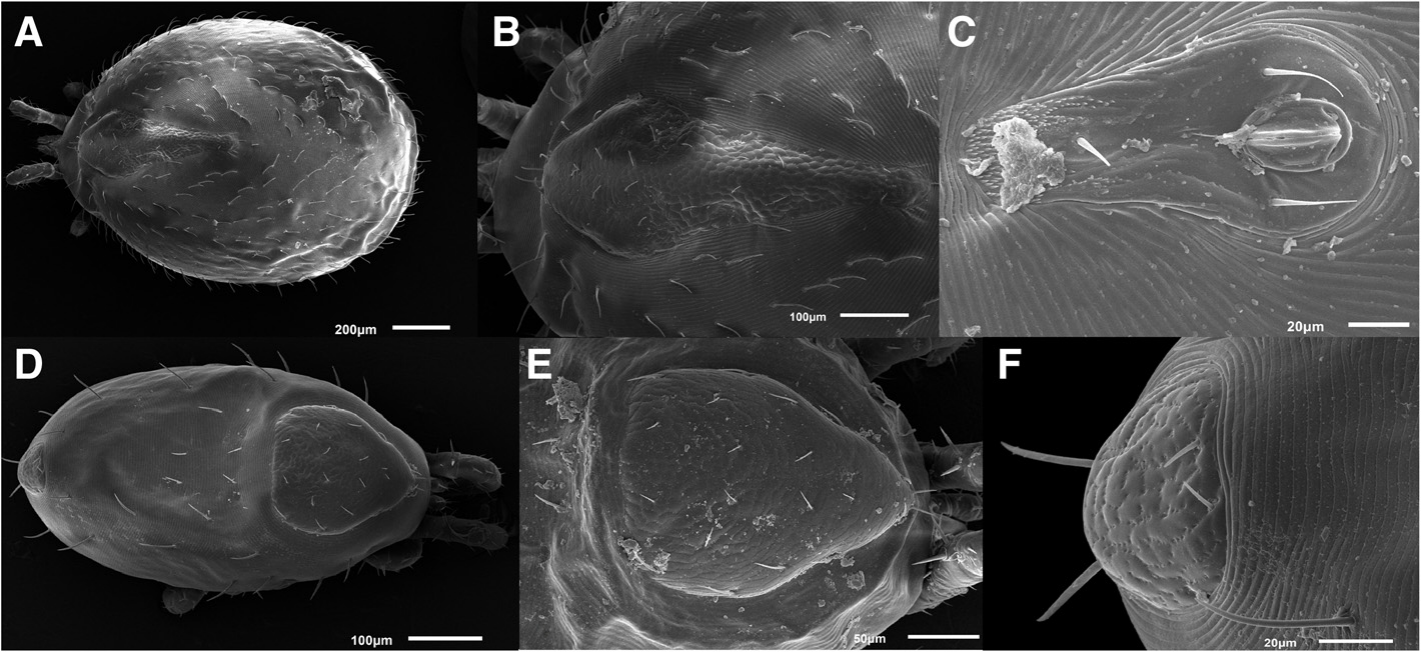
***Ophionyssus saurarum*** **A-C female of** ***O. saurarum*** **, D-F protonymph of** ***O. saurarum*** **; A and D dorsal view, B dorsal shield E podonotal shield C anal shield, F pygidial shield.**

Two species of ectoparasites on collected lizards; protonymphs and females of *Ophionyssus saurarum* mites (Figure 4) and larvae and nymphs of *I. ricinus* ticks, were identified. Prevalence of infestation with developmental stages (larvae, nymphs) of *I. ricinus* ticks ranged from 52.4% to 75.6%. Compared with ticks, mites were not collected in great quantities, so we did not reveal prevalence of infestation.

### Developmental stages in mites

**Figure 5:**
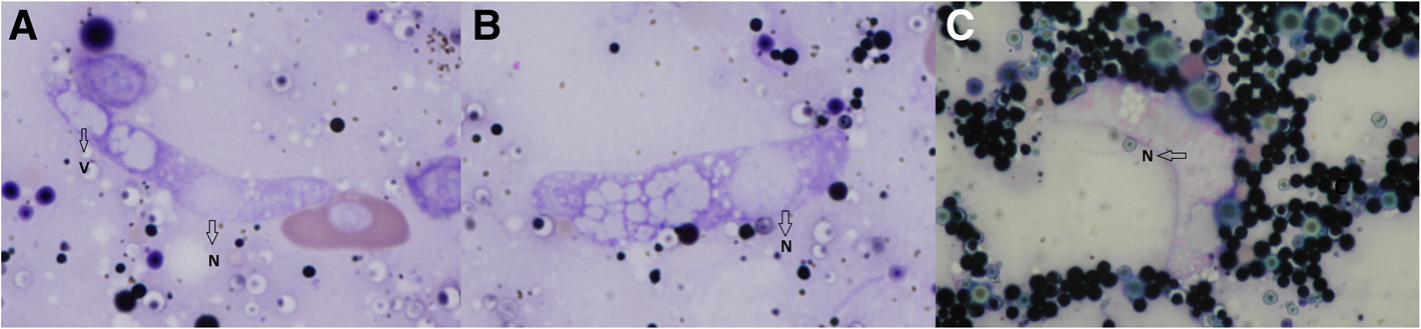
***Karyolysus*** **sp. found in** ***Ophionyssus*** **mites. A** sporokinete from the hemocoel of nymph of *Ophionyssus* found on wall lizard, Čabraď, **B** sporokinete from the clutch of eggs of *Ophionyssus* mite, **C** sporokinete from the smear of *Ophionyssus* mite found on green lizard, Tajba. Arrows indicates organelles: V = vacuoles, N = nucleus.

Smears of mites contained several free gamonts released from erythrocytes after blood sucking. Sporokinetes were found in the hemocoel of *Ophionyssus* mites (Figure 5, A and C) as well as from the clutch of eggs prepared immediately after oviposition (Figure 5, B). They had a pale blue cytoplasm with few but large vacuoles. The nucleus was located centrally or pericentrally with various appearances rather diffuse without a clear boundary (Figure 5).

### Molecular analysis

Only amplicons using the HEP300/HEP900 primers yielded usable DNA sequences of approximate 580 bps. Out of 210 DNA samples (54 *L. agilis*, 20 *L. agilis* ssp. *exigua*, one *L. agilis* ssp, *erythronota*, 95 *L. viridis*, one *L. viridis* ssp. *meridionalis*, 10 *L. trilineata* ssp. *dobrogica*, 12 *Z. vivipara* and 17 *P. muralis*), the fragment of *Karyolysus* sp. 18S rRNA was amplified in 64 (the prevalence of 30.5%) samples by PCR: 20 lizards from Hungary (*L. viridis*), 19 from Romania (one *L. agilis*, 11 *L. agilis* ssp. *exigua*, one *L. agilis* ssp, *erythronota*, one *L. viridis* and 5 *L. trilineata* ssp. *dobrogica*), 13 from Poland (10 *L. agilis* and 3 *Z. vivipara*) and 12 from Slovakia (2 *L. viridis* and 10 *P. muralis*) (Table 1). Eight isolates (five isolates of *Karyolysus* sp. 18S rRNA from lizards and 3 from ectoparasites) deposited in GenBank were used for phylogenetic analyses, including isolates of *K. lacazei* 18S rRNA from *L. viridis* (Hungary; KJ461943), *L. agilis* (Poland; KJ461940) and *L. trilineata* ssp. *dobrogica* (Romania; KJ461942); *K. latus* 18S rRNA from *P. muralis* (Slovakia; KJ461939) and *Karyolysus* sp. 18S rRNA from *Z. vivipara* (Poland; KJ461946). Besides lizards we also amplified the fragment of *Karyolysus* sp. 18S rRNA in *I. ricinus* and *O. saurarum* collected from lizards. For phylogenetic analyses three isolates of *Karyolysus* sp. 18S rRNA from these final hosts were used; from *O. saurarum* collected from *Z. vivipara* (Poland; KJ461945) and *L. viridis* (Hungary; KJ461944) and in a nymph of *I. ricinus* tick collected from *L. viridis* (Hungary; KJ461941). Details concerning GenBank accesion numbers are given in Additional file 1.

### Phylogenetic analysis

**Figure 6:**
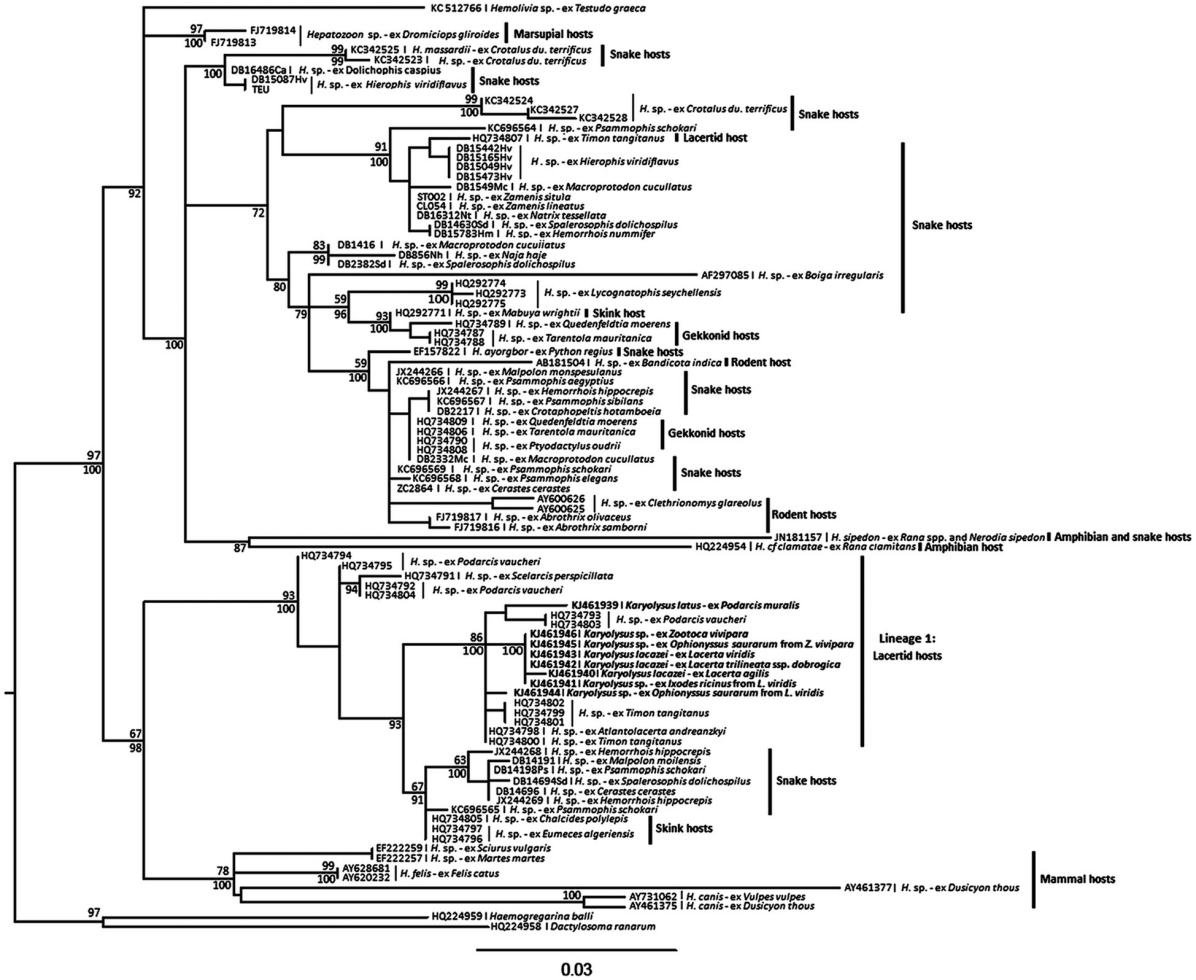
**Estimate of relationships of** ***Hepatozoon*** **and** ***Karyolysus*** **species based on 18S rRNA sequences.** Numbers above and below nodes indicates ML bootstrap support and Bayesian posterior probability values, respectively. The GenBank accession numbers and species of infected animals are in bold and resulted in existence of four haplotypes concerning to the same Lineage 1 (indicated behind vertical line).

Phylogenetic analyses (Bayesian method and Maximum Likelihood) gave the same overall estimate of phylogenetic patterns. Comparison of the eight isolates revealed the existence of four haplotypes, all part of the same lineage within sequences of parasites derived from North African lizards and snakes (Figure 6).

The first haplotype is represented by a parasite isolated from *P. muralis* (Slovakia, Krupina plateau). The second haplotype was identified from five isolates, including parasites from *Z. vivipara* (Poland), *L. viridis* (Hungary), *L. trilineata* ssp. *dobrogica* (Romania), *O. saurarum* collected from *Z. vivipara* (Poland) and *I. ricinus* from *L. viridis* (Hungary). The third haplotype is represented by parasite from *L. agilis* (Poland). The first, the second and the third haplotypes are closely related to parasites found in *P. vaucheri* from North Africa. Finally, the fourth haplotype is represented by the DNA sequence of the parasite isolated from *O. saurarum* from *L. viridis* (Hungary) and related to African parasites from *T. tangitanus* and *Atlantolacerta andreanskyi*.

**Figure 7:**
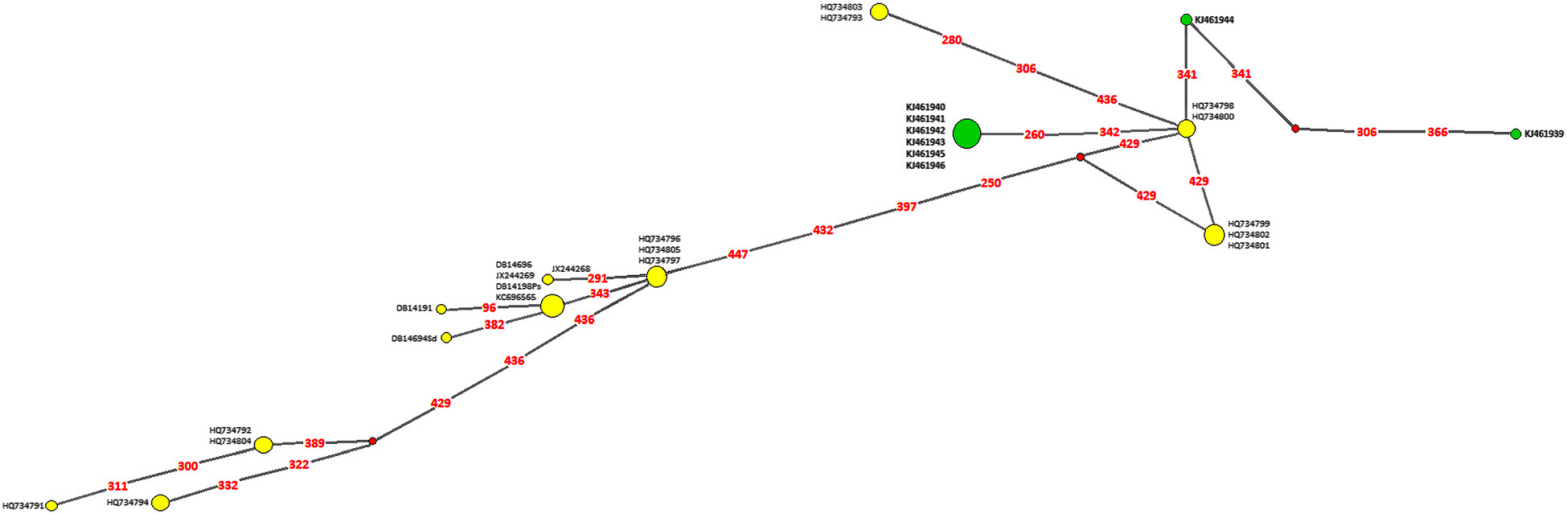
**Median-Joining Network analysis of lineage 1, using 455 bp 18S rRNA gene sequences.** *Karyolysus* sp. haplotypes obtained in this study are in bold with green coloured nodes.

Within the lineage 1, containing our isolates analyzed by Median-Joining Network, the majority of haplotypes obtained in this study (*Z. vivipara*, *L. viridis*, *L. trilineata* ssp. *trilineata*, *O. saurarum* from *Z. vivipara*, *I. ricinus* from *L. viridis* and *L. agilis*) formed a group, whereas *Karyolysus* parasites from *P. muralis* and *O. saurarum* from *L. viridis* were slightly genetically distinct (Figure 7). However, there was no association between the two morphologically identified species and the genetic relationships.

## Discussion and conclusions

Apicomplexan blood parasites represent a group of uni- and intra-cellular parasites, which can parasitize various species of animals with a worldwide distribution. We have only limited information about the presence of blood parasites in European reptiles, while even less is known about their molecular characterization. To the best of our knowledge this study represents the first assessment of genetic diversity of these parasites found in Central-Eastern European lizards.

Examined lizards were collected from twenty localities from four European countries (Hungary, Poland, Slovakia and Romania). Although we have scarce information about the prevalence of blood parasites found in reptiles from Poland and Romania, molecular detection of these parasites from European reptiles, collected in the above-mentioned countries, has never been performed.

Previously there was only information about the presence of blood parasites in *L. viridis, L. agilis agilis*, *L. a. chersonensis*, *Z. vivipara*, *E. orbicularis* and *Testudo graeca ibera* [2,9]. In Hungary, blood parasites of reptiles have been studied only on a small population of green lizards (25 individuals) [15]. In this study we identified haplotypes of blood parasites from all studied countries for the first time.

Overall prevalence of blood parasites in blood smears was 22.3%, but prevalence of infection between localities varied. We examined 13 individuals from Poland, and parasites were found in three animals (prevalence of 23.1%). Although representative sampling was quite low, results are comparable with 29.4% prevalence detected by Majláthová *et al.* [9]. For lizard species from Romania, parasites were observed in 10 individuals from 42 examined (prevalence of 23.8%), which is quite low in comparison with prevalence of 60.71% and 100% respectively, detected by Mihalca et al. [2]. A total of 84 green lizards (*L. viridis*) from Hungary were examined with 54 smears found to contain blood parasites (prevalence of 64.3%) in comparison with 96% prevalence found by Molnár et al. [15].

Although we examined reptiles from twelve different habitat types in Slovakia, blood parasites were detected only in four localities: Tajba, Krupinská planina, Fiľakovo castle ruins and near the water basin Ružín. These areas are localized mainly in the Southern part of Slovakia, where we can assume higher temperature during the year, which may be important for parasite development [4]. One exception represents the southernmost studied locality (Burda), where parasites were not found. On the other hand, Odolanów (Poland) is situated to the north of Slovakia, but the prevalence of blood parasites in the studied reptiles was relatively high [9]. Besides temperature as one of the key factors for the variable occurrence of blood parasites at our localities, altitude may also be important. Areas in Slovakia with presence of blood parasites are localized between 100 – 320 m. a. s. l., as well as areas in Poland (110 m. a. s. l.) and Hungary (124 and 211 m. a. s. l., respectively), whereas in reptiles from Romania from lower altitude were infected (15 – 200 m. a. s. l.). Other studies from Slovakia are situated between 380 – 1500 m. a. s. l., where conditions are probably less favorable for parasite development [4], although we observed the presence of ectoparasites on reptiles collected from the same locality. Contrarily, blood parasites found in lizards from southern part of Europe at higher altitudes from 650 – 2,200 m. a. s. l. have been detected [3,12-14,16-18].

Smears of mites collected from lizards contained several free gamonts released from erythrocytes after bloodsucking, and moreover sporokinetes were also found in the smear preparations from the mite eggs. The same results were observed only twice before, in mites collected from Scandinavian lizards [4,5]. These results showed that mites of *Ophionyssus* sp. serve as vectors for *Karyolysus* sp. in Europe, as demonstrated by experimental transmission and finding of sporokinets in mite’s eggs, which confirmed the presence of *Karyolysus* sp., because this genus of blood parasite is characterized by transstadial and transovarial transmission [5]. Except for this study, only the reptile intermediate hosts have been examined in Europe by microscopic observations, mainly in the Mediterranean region [11-14,16]. One of the life cycle differences between *Karyolysus, Hepatozoon* and *Hemolivia* is transovarial transmission which was not described in *Hepatozoon* or *Hemolivia* but occurs in *Karyolysus* and we observed it as well.

Phylogenetic analysis showed that isolates obtained in this study fall within the same lineage with sequences of parasites originating from North African reptiles, which were identified as *Hepatozoon* sp. [31]. The lineage containing isolates obtained in this study differs on one hand from sequences of *Hepatozoon* parasites isolated from African geckos (*Ptyodactylus* sp., *Quedenfeldtia* sp. and *Tarentola* sp.), snakes (*Python regius*, *Boiga irregularis* and *Lycognatophis seychellensis*) and rodents (*Clethrionomys* sp. and *Abrothrix* sp.) from Chile, Spain and Thailand, and on the other hand from *Hepatozoon* isolates primarily from dogs and cats. Previously, *Karyolysus* has never been characterized by molecular methods [2-5]; identification of *Karyolysus* in reptile species was based on microscopic methods only. Current taxonomy is greatly complicated by the identification of parasites by a limited number of morphological attributes, which are clearly not consistent. Thus the genus *Karyolysus*, as the Greek name implies, was first proposed for hemogregarines that distort the host cell nucleus. However, “karyolysing hemagregarines” such as ones identified in Algerian lacertids, *Timon* sp., are now classified as *Hepatozoon curvirostris*, and not *Karyolysus*. Not only this, but apparent *Karyolyus* species identified in this study are genetically related to forms from Iberian and North African reptiles in which deformation of the host nucleus was not reported [18]. At the same time other characters are applied haphazardly for identification of parasites - gamonts are identified as *Karyolysus* due to the vertebrate host they are found in, or “probably” to *Hepatozoon* when the same hosts, *P. muralis*, are heavily infected with ticks for example [3]. Furthermore the same genetic lineage of parasites is found in this study in both ticks and mites. It is clear therefore that no simple alteration to taxonomy will resolve the issue. Identifying the whole genetic lineage (1) as *Karyolysus* would mix forms that apparently both do and do not distort the host nucleus. However, any other arrangement would make *Hepatozoon* paraphyletic. Since new lineages are regularly being identified, for example in birds [32], or caecilians [20] it also seems premature to rearrange the nomenclature, since new discoveries will almost certainly alter our understanding of evolutionary relationships of these parasites [33]. A similar situation arises regarding the genus *Hemolivia*, which also appears to be part of the same major group with *Hepatozoon* and *Karyolysus*, and for which relationships vary depending on the out-groups employed [21,34]. Although the sequences used in this study are quite short (584 bp), this issue is unlikely to be resolved with a longer fragment of 18S rRNA, since [18] already demonstrated that estimates of relationships based on this short fragment were the same as those based on the longer fragment employed in some other studies. However, the slow-evolving nature of the marker may be part of the problem in observing differences between the two morphologically identified species. Faster evolving genes may be necessary to disentangle relationships at the species level.

Although the result of phylogenetic analysis placed *Karyolysus* sp. isolates obtained in this study within *Hepatozoon* making this genus paraphyletic [19,21,35,36], we can observe differences in biology of these two genera of parasites. *Hepatozoon* is transmitted via ingestion of a wide spectrum of invertebrates (ixodid and argasid ticks, triatomid bugs, leeches, flies, sucking lice, fleas, sandflies and mosquitoes) [37] and is characterized by polysporocystic oocysts formed in hemocoel of the abdomen, thorax or within the head of final host [1,37]. Moreover transovarial transmission of the *Hepatozoon* in definitive invertebrate hosts has never been demonstrated [38-40]. On the contrary, the only final invertebrate host identified for the *Karyolysus* sp. is represented by mites of the genus *Opionyssus* [1,4,5]. *Karyolysus* is also characterized by sporozoites within oocysts localized in the gut cells of the final host [1,19] as well as transovarial transmission within the final host have also been observed [1,4,5]. Based on the results we can conclude that molecular data available are insufficient to reveal actual position of *Karyolysus* sp. with/within *Hepatozoon* sp.

This work represents the first molecular insight to the phylogeny of *Karyolysus* sp. found in studied reptile species collected in various localities of Central-Eastern Europe. Previously, species of *Karyolysus* were detected primarily using morphological characteristics of gamonts found in infected reptile hosts. Our study indicates this is unsatisfactory, and that the incorporation of molecular data has clear advantages. The combined approach used in this study could reveal further discrepancies in the actual classification, and we suggest is enlarged to include additional geographic regions and other potential reptile intermediate hosts of these poorly-known parasites.

### Ethical approval for animal use

Capturing lizards and sample collection were carried out with official permission from the Middle Danube Valley Inspectorate for Environmental Protection, Nature Conservation and Water Management (Hungary), 6103/2007-2.1 and 5498/2011-2.2 issued by the Ministry of Environment of the Slovak Republic, and 12/2007 issued by the local ethics committee for animal studies in Poznań (Poland).

## Additional file

Additional file 1:
**The GenBank accession numbers of** ***Karyolysus*** **samples obtained in this study.**
