# Supplementary material for: Morphological and molecular characterization of Karyolysus – a neglected but common parasite infecting some European lizards
Source: Parasit Vectors. 2014 Dec 10;7:555. doi: 10.1186/s13071-014-0555-x (PMC4298996; doi:10.1186/s13071-014-0555-x)
Supplement: Additional file 1: — The GenBank accession numbers of Karyolysus samples obtained in this study. [file 13071_2014_555_MOESM1_ESM.doc]

| Species | Country | Study area | Species of *Karyolysus* | GenBank  Accession number |
| --- | --- | --- | --- | --- |
| *Lacerta agilis* | Poland | Odolanów | *K. lacazei* | KJ461940 |
| *L.viridis* | Hungary | Gödöllő | *K. lacazei* | KJ461943 |
| *L. trilineata dobrogica* | Romania | Sacele | *K. lacazei* | KJ461942 |
| *Podarcis muralis* | Slovakia | Krupinská planina | *K. latus* | KJ461939 |
| *Zootoca vivipara* | Poland | Odolanów | *Karyolysus* sp. | KJ461946 |
| *Ophionyssus saurarum* from *Z. vivipara* | Poland | Odolanów | *Karyolysus* sp. | KJ461945 |
| *O. saurarum* from *L. viridis* | Hungary | Gödöllő | *Karyolysus* sp. | KJ461944 |
| *Ixodes ricinus* from *L. viridis* | Hungary | Gödöllő | *Karyolysus* sp. | KJ461941 |

The GenBank accession numbers of *Karyolysus* samples obtained in this study.
